# Enhancing precision medicine: a nomogram for predicting platinum resistance in epithelial ovarian cancer

**DOI:** 10.1186/s12957-024-03359-9

**Published:** 2024-03-21

**Authors:** Ruyue Li, Zhuo Xiong, Yuan Ma, Yongmei Li, Yu’e Yang, Shaohan Ma, Chunfang Ha

**Affiliations:** 1 Department of Gynecology, General Hospital of Ningxia Medical University, Yinchuan, Ningxia People’s Republic of China; 2 Ningxia Medical University, Yinchuan, Ningxia People’s Republic of China; 3 Department of Gynecologic Oncology, General Hospital of Ningxia Medical University, Yinchuan, Ningxia People’s Republic of China; 4 Key Laboratory of Reproduction and Genetic of Ningxia Hui Autonomous Region, Key Laboratory of Fertility Preservation and Maintenance of Ningxia Medical University and Ministry of Education of China, Department of Histology and Embryology in, Ningxia Medical University, Yinchuan, Ningxia People’s Republic of China

**Keywords:** Epithelial ovarian cancer, Clinical features, Platinum resistance genes, Predictive modeling, Nomogram

## Abstract

**Background:**

This study aimed to develop a novel nomogram that can accurately estimate platinum resistance to enhance precision medicine in epithelial ovarian cancer(EOC).

**Methods:**

EOC patients who received primary therapy at the General Hospital of Ningxia Medical University between January 31, 2019, and June 30, 2021 were included. The LASSO analysis was utilized to screen the variables which contained clinical features and platinum-resistance gene immunohistochemistry scores. A nomogram was created after the logistic regression analysis to develop the prediction model. The consistency index (C-index), calibration curve, receiver operating characteristic (ROC) curve, and decision curve analysis (DCA) were used to assess the nomogram’s performance.

**Results:**

The logistic regression analysis created a prediction model based on 11 factors filtered down by LASSO regression. As predictors, the immunohistochemical scores of CXLC1, CXCL2, IL6, ABCC1, LRP, BCL2, vascular tumor thrombus, ascites cancer cells, maximum tumor diameter, neoadjuvant chemotherapy, and HE4 were employed. The C-index of the nomogram was found to be 0.975. The nomogram’s specificity is 95.35% and its sensitivity, with a cut-off value of 165.6, is 92.59%, as seen by the ROC curve. After the nomogram was externally validated in the test cohort, the coincidence rate was determined to be 84%, and the ROC curve indicated that the nomogram’s AUC was 0.949.

**Conclusion:**

A nomogram containing clinical characteristics and platinum gene IHC scores was developed and validated to predict the risk of EOC platinum resistance.

## Background

About 85% of patients with epithelial ovarian cancer will achieve clinical remission after surgery combined with chemotherapy [1]. However, a few patients may resist platinum-based chemotherapy due to differences in tumor biology, resulting in an inferior clinical prognosis [2]. Some studies have concentrated on the differences in clinical characteristics, such as tumor markers CA125 and HE4, between platinum-resistant and platinum-sensitive patients to seek clinical characteristics with predictive value [3]. These indicators can usually be obtained through routine examination and surgical pathology of patients without additional invasive examination and without increasing the economic burden of patients, but applying them alone has limited predictive value. Emil Lou et al. tried to use tumor-stroma proportion to predict platinum resistance of ovarian cancer, but the prediction specificity was low [4]. Other literature reviews have suggested that essential signaling molecules involved in the cellular mechanism of platinum resistance in ovarian cancer can be used as markers for platinum resistance prediction [5–8]. Jesus Gonzalez Bosquet et al. examined some platinum resistance genes as predictive models to predict the possibility of platinum resistance in EOC patients, and the AUC of the prediction model with 34 genes was 80% after optimization [9]. As with other methods that use immune genes and lncRNAs to predict platinum resistance [10, 11], the predictive value of these methods is not particularly high, and the economic cost and quality standards of genetic testing greatly affect their application. So, how the predictive value of these platinum resistance-associated signaling molecules and clinical characteristics can be applied to clinical work needs to be further explored.

In this study, clinical characteristics combined with immunohistochemical(IHC) score of platinum resistance gene to build a platinum resistance prediction model for epithelial ovarian cancer, hoping to more accurately identify the patients who can not benefit from the first-line platinum-based chemotherapy regimen and provide some reference value for the development of personalized chemotherapy regimen for patients with epithelial ovarian cancer.

## Methods

### Research object and organization

Patients with epithelial ovarian cancer treated with standard initial treatment (satisfactory cytoreductive surgery combined with chemotherapy) in the General Hospital of Ningxia Medical University from January 31, 2019, to June 30, 2021, were selected as sample sources. Inclusive criteria: (1) epithelial ovarian cancer diagnosed by surgery and pathology, (2) receiving standard initial treatment regimen (satisfactory cytoreductive surgery combined with chemotherapy), (3) the general data of the patients were complete, and the routine examination, serum CA 125, HE4 indexes and imaging examination were recorded regularly. Exclusion criteria:(1) combined with other malignant diseases, digestive tract diseases, acute and chronic inflammation; (2) not receiving platinum-containing chemotherapy, (3)follow-up data were incomplete.

According to the above criteria, 95 samples were collected for model construction, and 70 patients before December 31, 2020, as a training group, after 25 samples as a test set. All patients who participated in the study signed informed consent. All enrolled patients were divided into the platinum-resistant group(Res-group) and platinum-sensitive group(Sen-group) according to the following diagnostic criteria: patients in the platinum-resistant group include those who are platinum-resistant or platinum-refractory, platinum-resistant refers to those who respond to initial chemotherapy but progress or relapse within six months of completing chemotherapy, and platinum-refractory refers to those who do not respond to initial chemotherapy, such as stable tumors or tumors that progress, including those that progress within four weeks of chemotherapy. Platinum-sensitive refers to those who respond to initial chemotherapy but progress or relapse after six months of completing chemotherapy.

### Clinical characteristics, tissue collection and patient follow-up

This study retrospectively collected the medical records of patients with epithelial ovarian cancer and collected the general conditions, examination results, treatment plans, and other data of patients through the electronic medical record system. Tumor tissue sections were obtained from the hospital pathology department for IHC.

The enrolled patients were followed up by telephone and medical record data inquiry until February 2022. The median follow-up time was 27.0 (16.0$\sim$33.8) months. The follow-up interval of patients was every 2∼4 months in the first two years, every 4–6 months in the third five years, and every 6∼12 months after five years. Each follow-up included asking about symptoms, physical examination, tumor markers, and chest and abdomen CT or MRI examination.

### Screening of platinum resistance genes

The GEO database (https://www.ncbi.nlm.nih.gov/geo/) was searched with the keywords “Ovarian cancer” and “Drug resistance”, and the screening conditions were: The species is “Homo sapiens”, the attribute is “Tissue”, and the study type is “Expression profiling by array”. Two datasets with significantly different genes (DEGs) are obtained: GSE45553 and GSE28739. The GSE45553 dataset is based on the GPL6244 data platform, including four platinum-resistant and four platinum-sensitive tissues. The GSE28739 dataset is based on the GPL7264 data platform, including 20 platinum-resistant and 30 platinum-sensitive tissues. The batch correction ratio is used to screen DEGs with “sva” and “limma” in R software, and the volcano diagram is drawn. The screening criteria of DEGs are set as follows: |log FC|> 1.0, *p* < 0.05. Heat map of DEGs was drawn by “heatmap” of R software, “clusterprofiler”,“org. db “,” richplot, “and” ggplot2 “were used to annotate the differential genes in Gene Ontology (GO), and Kyoto Encyclopedia of genes and genomes(KEGG) functional enrichment analysis was performed to p value < 0.05 was used as the inclusion criteria. Significant difference genes were analyzed by STRING online database (https://cn.string-db.org/) to analyze the protein-protein interaction (PPI) network of gene coding, and then the functional modules were constructed by Cytoscape software MCODE plug-in clustering, and the genes in the sub-network with the highest score were selected as hub genes.

In addition, this study screened nine classical platinum resistance-related genes to participate in the variable screening of the prediction model through the literature method, including the most critical intracellular transporter of platinum drugs, copper transporter 1 (CTR 1), whose expression level affects the chemotherapy effectiveness of platinum drugs Drug efflux-related genes: ABCC 1 transporter is also known as multidrug resistance-associated protein 1 (MRP 1), P-glycoprotein (P-gp), breast cancer resistance protein (BCRP), lung resistance-related protein (LRP), apoptosis-related genes such as p53, B-cell lymphoma-2 (Bcl-2), Survivin, DNA damage repair-related genes such as Resect and repair cross complementation one protein (ERCC 1) [12, 13].

### Immunohistochemistry

The slices were washed with environment-friendly dewaxing solution I 10 min, environment-friendly dewaxing solution II 10 min, environment-friendly dewaxing solution III 10 min, absolute ethanol I 5 min, absolute ethanol II 5 min, absolute ethanol III 5 min and distilled water in turn. The antigen was recovered by heating. Endogenous peroxidase was blocked with 3% hydrogen peroxide. The slides were blocked with 3% BSA for 30 min at room temperature. The primary antibody prepared by PBS was dropped on the slides (SOCS 3 Polyclonal antibody, CEBPB Polyclonal antibody, IL-1 Beta Polyclonal antibody, CXCL 1 Polyclonal antibody, LIF Polyclonal antibody, P glycoprotein Polyclonal antibody, BCRP, ABCG 2 Polyclonal antibody, MVP/LRP Polyclonal antibody, human BCL 2 Polyclonal antibody, P53 Polyclonal antibody, SURVIVIN Polyclonal antibody, ERCC 1 Polyclonal antibody was from Wuhan Sanying Biotechnology, China, and SLC 31 A1/CTR 1 Antibody, ABCC 1 Antibody was from Affinity Biosciences, USA). The sections were placed in a wet box and incubated at 4 ° C for 12 h. Sections were rinsed three times with PBS and incubated with a secondary antibody (HRP-labeled rabbit anti-goat IgG, GB 23,204, Servicebio) for 30 min at room temperature, immunostained with 3∼30 diaminobenzidine tetrahydrochloride (DAB), and tissue samples were counterstained with hematoxylin. Sections were examined by light microscopy.

Image J software analyzed immunohistochemical pictures’ average optical density value (AOD). In addition, in the process of model establishment, IHC results were automatically scored by the IHC Profiler plug-in [14], and the average gray value of positive cells (staining intensity) and percentage of positive area (staining area) were used as IHC measurement indicators to give four scores: High positive (3+), Positive (2+), Low Positive (1+) and Negative (0).

### Statistical method

GraphPad Prism8.0 software was used to analyze the data. The measurement data were compared by independent sample t-test. The comparison of count data rates was tested and analyzed, and the IHC score was used as an ordered variable by the Mann-Whitney test. Using R® (version 4.1.1) to establish and verify the prediction model, using LASSO regression analysis to screen out the variables, based on the Logistics regression analysis to establish the prediction model, using nomogram function to draw the nomogram. Calculate the consistency index (C-index) to evaluate the model’s predictive value. Using 1000 times of enhanced Bootsrap to draw the calibration curve. Draw the Receiver operating curve(ROC) to calculate the model cut-off value and sensitivity specificity and the decision curve to analyze and evaluate the model performance. In the external verification process of the test set, draw ROC to calculate AUC, calculate the prediction model concordance rate, and evaluate the model’s accuracy by the kappa test.

## Results

### Clinical characteristics of EOC patients related to platinum-resistant in the training cohort

In the training set, univariate analysis of clinical characteristics that might be associated with platinum-resistance in patients with epithelial ovarian cancer showed significant differences between platinum-sensitive and platinum-resistant patients in age, lymph node metastasis, vascular cancer thrombus, ascites cancer cells, neoadjuvant chemotherapy, and tumor marker HE4. However, the two groups had no significant differences in maximum tumor diameter, FIGO stage, histopathological type, grade, residual tumor size, and tumor marker CA125(Table 1). Considering their possible clinical significance, we still screened them as initial variables.

**Table 1:** Clinical characteristics of epithelial ovarian cancer patients in training cohort (*n* = 70)

Clinical features	Sen-group(*n* = 43) (n/%)/$\bar {x} \pm s$	Res-group(*n* = 27) (n/%)/$\bar {x} \pm s$	t/χ^2^	p
Age				
≤ 50 years	19(44.2)	5(18.5)	4.850	0.028
> 50years	24(55.8)	22(81.5)		
Maximum tumor diameter				
≤ 5 cm	13(30.2)	13(48.1)	2.280	0.131
> 5 cm	30(69.8)	14(51.9)		
FIGO stage				
I/II	12(27.9)	3(11.1)	2.779	0.096
III/IV	31(72.1)	24(88.9)		
Histopathological type				
serous	36(83.7)	25(92.6)	1.165	0.280
others	7(16.3)	2(7.4)		
Tumor grade				
G3	32(74.4)	23(85.2)	1.142	0.285
G1-G2	11(25.6)	4(14.8)		
Lymph node metastasis				
unresected	18(41.9)	13(48.1)	6.534	0.038
no	14(32.6)	2(7.4)		
yes	11(25.6)	12(44.4)		
Vascular cancer thrombus				
no	32(74.4)	10(37.0)	9.657	0.002
yes	11(25.6)	17(63.0)		
Ascites cancer cell				
no	22(51.2)	4(14.8)	9.386	0.002
yes	21(48.8)	23(85.2)		
Residual tumor size^*^				
R0	27(62.8)	11(40.7)	4.717	0.095
R1	13(30.2)	10(37.0)		
R2	3(7.0)	6(22.2)		
Neoadjuvant chemotherapy				
no	36(83.7)	11(40.7)	13.888	0.000
yes	7(16.3)	16(59.3)		
CA125(U/L)	874.35 ± 1268.85	1558.4 ± 1632.69	2.655	0.054
HE4(pmol/L)	313.06 ± 263.33	606.15 ± 356.95	-3.945	0.000

*p* < 0.05 were considered statistically signifcant

*Residual tumor size: R0 = total tumor resection, R1 = microscopic residual, R2 = gross residual

### Screening and validation of platinum resistance genes in epithelial ovarian cancer

**Fig. 1:**
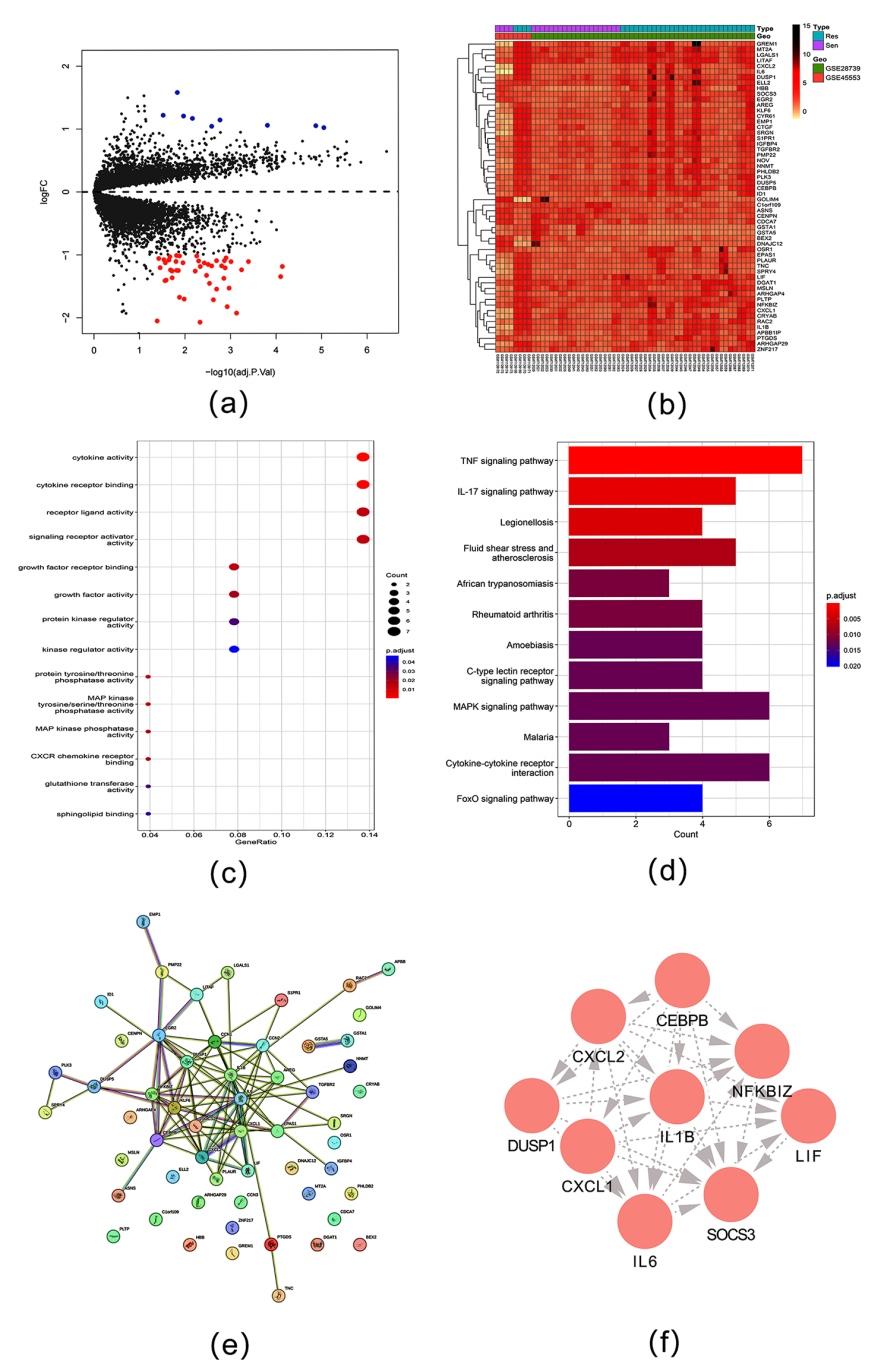
Screening of platinum resistance genes in epithelial ovarian cancer. (**a**) Differential gene heat map of GSE45553 and GSE28739 datasets, which conforms to |log FC|>1.0, *p* < 0.05). (**b**) Heat map of differentially expressed genes. (**c**) GO function enrichment of platinum resistance genes. (d) KEEG pathway enrichment of platinum resistance genes. (**e**) PPI network of 56 differentially expressed genes. (**f**) MCODE sub-network with the highest clustering score.

GEO database screening datasets GSE45,553 and GSE28,739 were batch corrected and finally screened 56 platinum resistance and significantly different genes in epithelial ovarian cancer, of which 47 were up-regulated and 9 were down-regulated (Fig. 1a). The fusion dataset’s differential expression gene heat map (Fig. 1b) visually presented the expression of significantly different genes. The GO function enrichment results of 56 genes significantly differentially expressed in tumor tissues of platinum-resistant and platinum-sensitive patients showed that their molecular functions were mainly expressed in cytokine activity, cytokine receptor binding, receptor-ligand activity, signal receptor activator activity(Fig. 1c). The KEEG signaling pathway enrichment analysis showed that the differentially expressed genes mainly enriched in the TNF signaling pathway, IL-17 signaling pathway, cytokine-cytokine receptor interaction (Fig. 1d).

Construction of PPI network of 56 significantly different genes through STRING online database (Fig. 1e). Then the PPI network was imported into Cytoscape software, and the highest scoring subnetwork was selected through MCODE plug-in clustering analysis ((Fig. 1f)) as hub genes, including Suppressor of Cytokine Signaling 3 (SOCS3), CCAAT/enhancer binding protein CCAAT/ enhancer binding protein (CEBP), interleukin-1β (ILB1), chemokines (C-X-C Motif) Ligand 1 (CXCL1), chemokine (C-X-C motif) ligand 2 (CXCL2), interleukin 6 (IL6), Dual specificity protein phosphatase 1 (DUSP1), ζ nuclear factor of Kappa light polypeptide gene enhancer in B-cells inhibitor(NFKBIZ), Leukemia inhibitory factor (LIF).

**Fig. 2:**
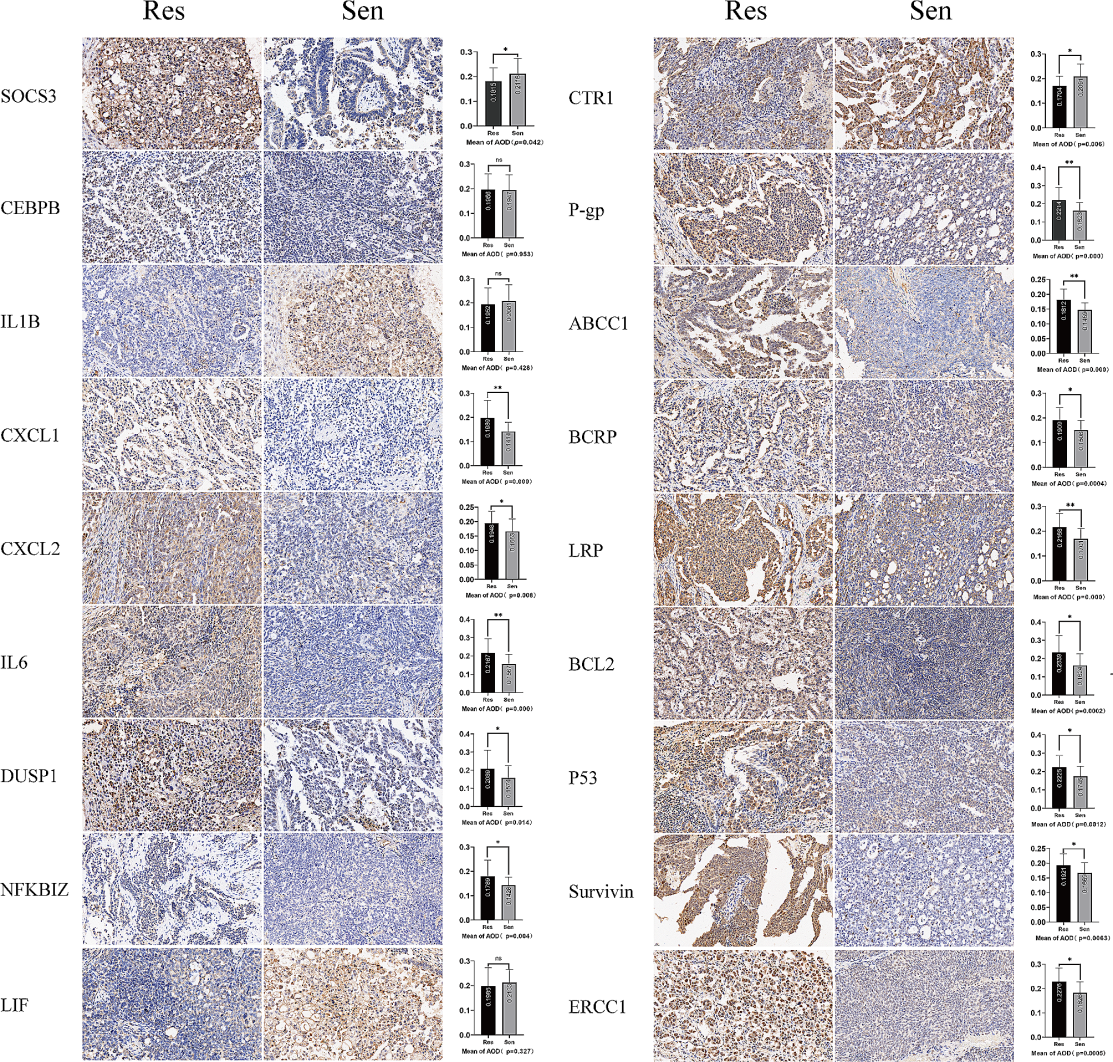
Immunohistochemical verification of platinum resistance gene in epithelial ovarian cancer. (Magnification: ×400, quantitative analysis AOD, ^*^p < 0.05)

Immunohistochemical detection(Fig. 2) results of 5 hub genes in the two groups were consistent with gene sequencing. CXCL1 was mainly localized in the cytoplasm, and its expression in the platinum-resistant group was significantly higher than in a platinum-sensitive group (*p* < 0.001). The expression of CXCL2 in the platinum-resistant group was significantly higher than in the platinum-sensitive group (*p* = 0.008). The expression of IL6 in the platinum-resistant group was significantly higher than that in the platinum-sensitive group (*p* < 0.001). DUSP1 was mainly expressed in the cytoplasm, and the expression in the platinum-resistant group was significantly higher than in the platinum-sensitive group (*p* = 0.014). The expression of NFKBIZ was mainly in the nucleus of cells, and the expression in the platinum-resistant group was significantly higher than that in the platinum-sensitive group (*p* = 0.004).

The nine platinum resistance genes screened by the literature method were further verified by immunohistochemistry (Fig. 2), and the results were consistent with the literature results. The expression of CTR1 in tumor tissues of patients with platinum resistance was lower than that of patients with platinum sensitivity, and the difference was statistically significant(*p* = 0.006). The expressions of P-gp, ABCC1, BCRP, LRP, BCL2, P53, Survivin, and ERCC1 in the platinum-resistant group were significantly higher than those in the platinum-sensitive group(*p* < 0.05). Further IHC scores for 14 genes are shown in Table 2, and 14 genes have differences in IHC scores between platinum-resistant and platinum-sensitive groups(*p* < 0.05).

**Table 2:** Platinum resistance gene IHC score* in epithelial ovarian cancer

IHC score		Sen-group(n/%)	Res-group(n/%)	p
CXCL1	0	39(90.70)	9(33.3)	0.000
	1+	4(9.3)	16(59.3)	
	2+	0(0)	2(7.4)	
CXCL2	0	33(76.7)	7(25.9)	0.000
	1+	10(23.3)	17(63.0)	
	2+	0(0)	3(11.1)	
IL6	0	32(74.4)	4(14.8)	0.000
	1+	11(25.6)	14(51.9)	
	2+	0(0)	9(33.3)	
DUSP1	0	35(81.4)	14(51.9)	0.006
	1+	8(18.6)	10(37)	
	2+	0(0)	3(11.1)	
NFKBIZ	0	38(88.4)	15(55.6)	0.002
	1+	5(11.6)	12(44.4)	
CTR1	0	13(30.2)	18(66.7)	0.000
	1+	18(41.9)	9(33.3)	
	2+	12(27.9)	0(0)	
P-gp	0	33(76.7)	10(37.0)	0.000
	1+	10(23.3)	12(44.4)	
	2+	0(0)	5(18.6)	
ABCC1	0	40(93.0)	15(55.6)	0.000
	1+	3(7.0)	12(44.4)	
BCRP	0	35(81.4)	10(37.0)	0.000
	1+	8(18.6)	15(55.6)	
	2+	0(0)	2(7.4)	
LRP	0	32(74.4)	7(26.0)	0.000
	1+	11(25.6)	10(37.0)	
	2+	0(0)	10(37.0)	
BCL2	0	36(83.7)	8(29.6)	0.000
	1+	7(16.3)	16(59.3)	
	2+	0(0)	3(11.1)	
P53	0	36(83.7)	11(40.7)	0.000
	1+	7(16.3)	14(51.9)	
	2+	0(0)	2(7.4)	
Survivin	0	35(81.4)	15(55.6)	0.018
	1+	8(18.6)	11(40.7)	
	2+	0(0)	1(3.7)	
ERCC1	0	33(76.7)	7(25.9)	0.000
	1+	10(23.7)	15(55.6)	
	2+	0(0)	5(18.5)	

*IHC score: Using the IHC Profiler, the average gray value (staining intensity) and the percentage of the positive area (staining area) of the positive cells are taken together as IHC measurement indicators to give four scores: High positive (3+), Positive (2+), Low Positive (1+) and Negative (0)

*p* < 0.05were considered statistically signifcant

### Construction and evaluation of platinum resistance prediction nomogram in epithelial ovarian cancer

**Fig. 3:**
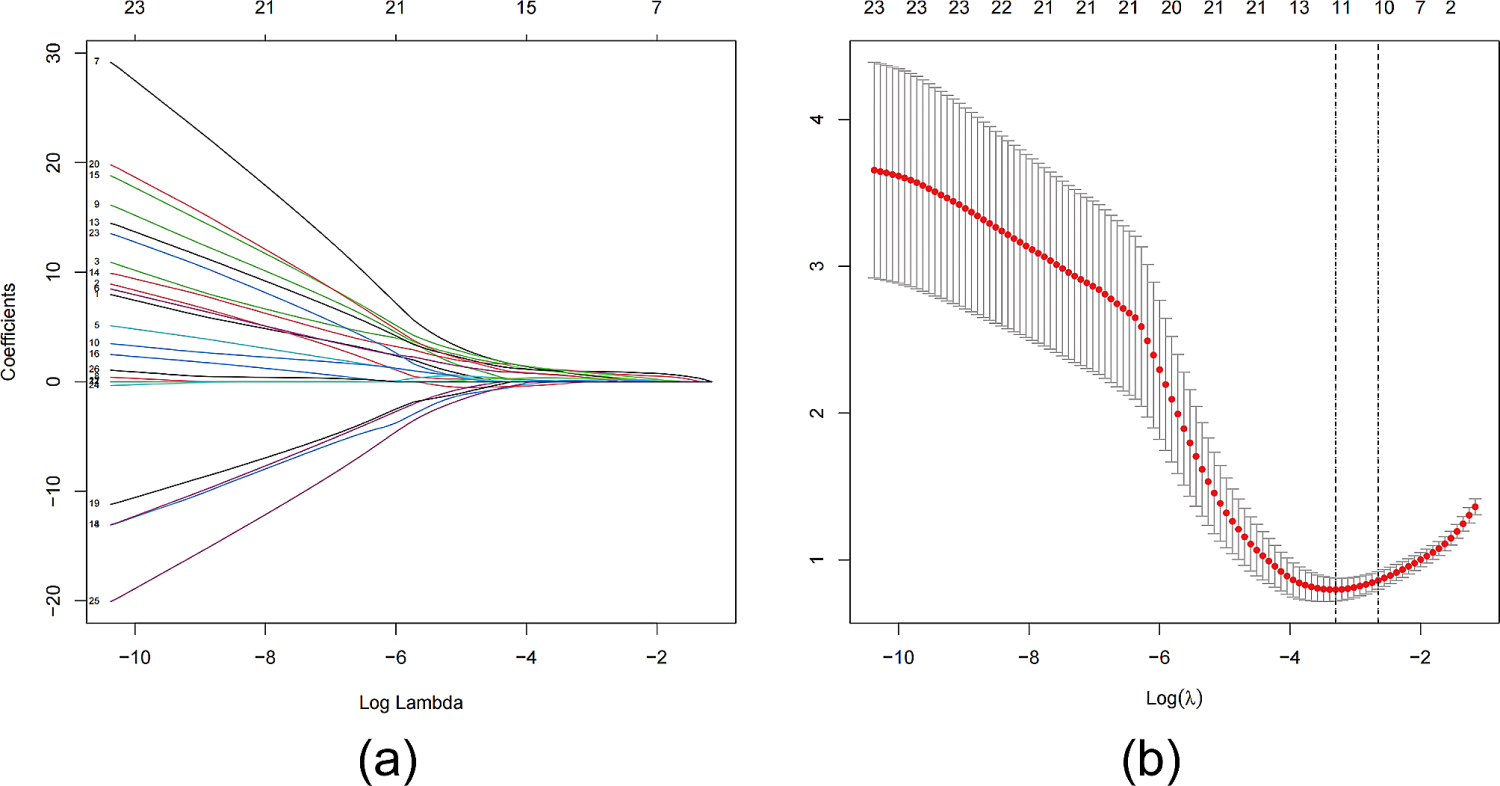
LASSO regression screening variables. (**a**) Variation characteristics of LASSO regression coefficients. (**b**) LASSO regression through cross-validation method to screen the most appropriate parameter lambda value, the left dotted line represents the minimum target parameter mean lambda = 0.0368666, in this case, the model into 11 variables will perform best.

**Fig. 4:**
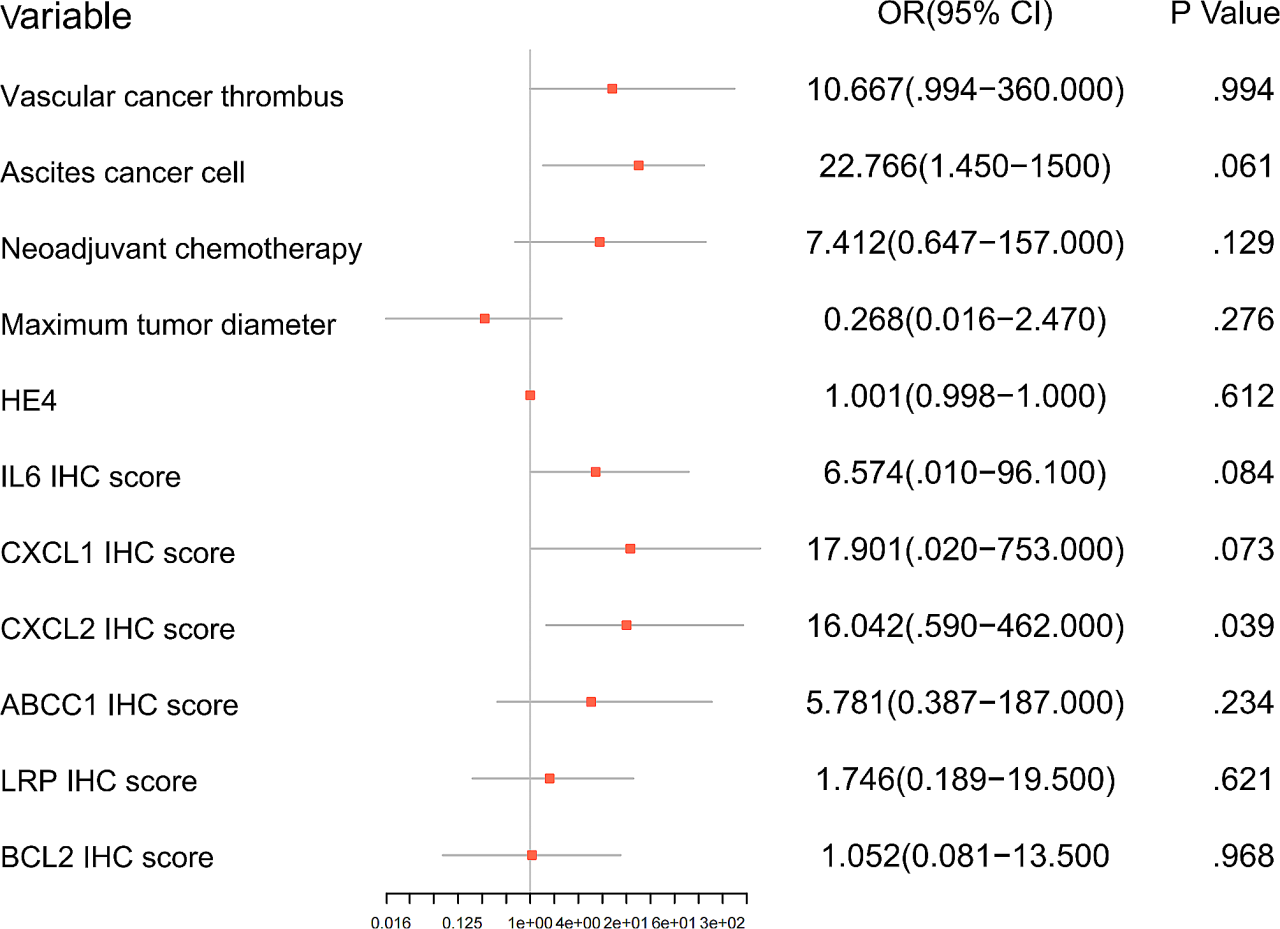
Results of Logistic regression analysis for 11 variables.

**Fig. 5:**
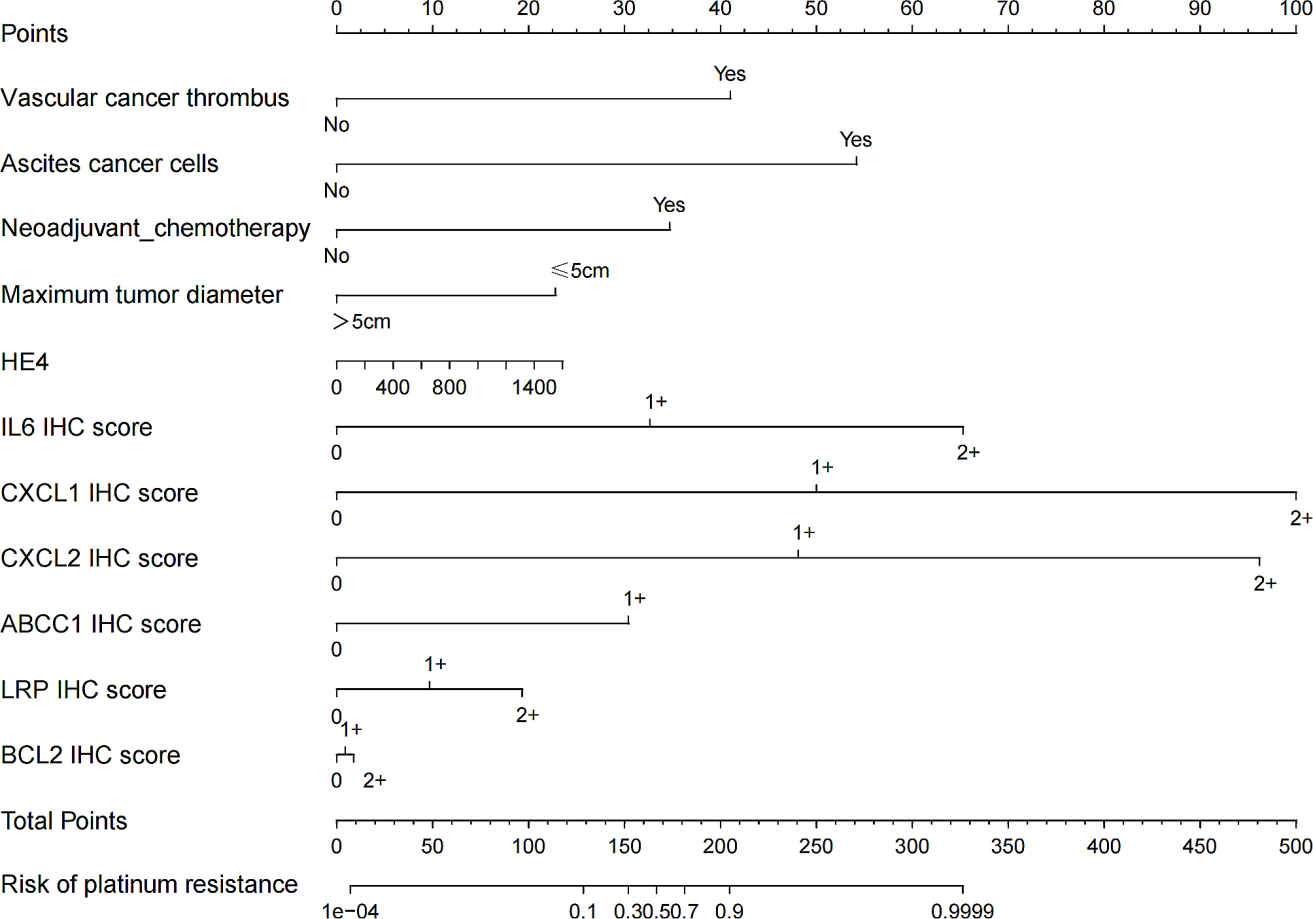
Nomogram of platinum resistance prediction in epithelial ovarian cancer.

Considering the small sample size of the training cohort and the large number of variables, we screened 12 clinical features and 14 platinum resistance gene IHC scores by LASSO regression analysis, with platinum resistance of epithelial ovarian cancer as the dependent variable, when lambda was 0.0368666 (Fig. 3a), indicating that the model achieved the best performance in the case of 11 risk factors (Fig. 3b). IHC scores of CXCL1、CXCL2、IL6、ABCC1、LRP、BCL2, vascular tumor thrombus, ascites cancer cells, maximum tumor diameter, neoadjuvant chemotherapy, and HE4 were included in the prediction model. The results of logistics regression analysis for these included prediction models are shown in Fig. 4, and a nomogram for predicting platinum resistance was further constructed (Fig. 5).

**Fig. 6:**
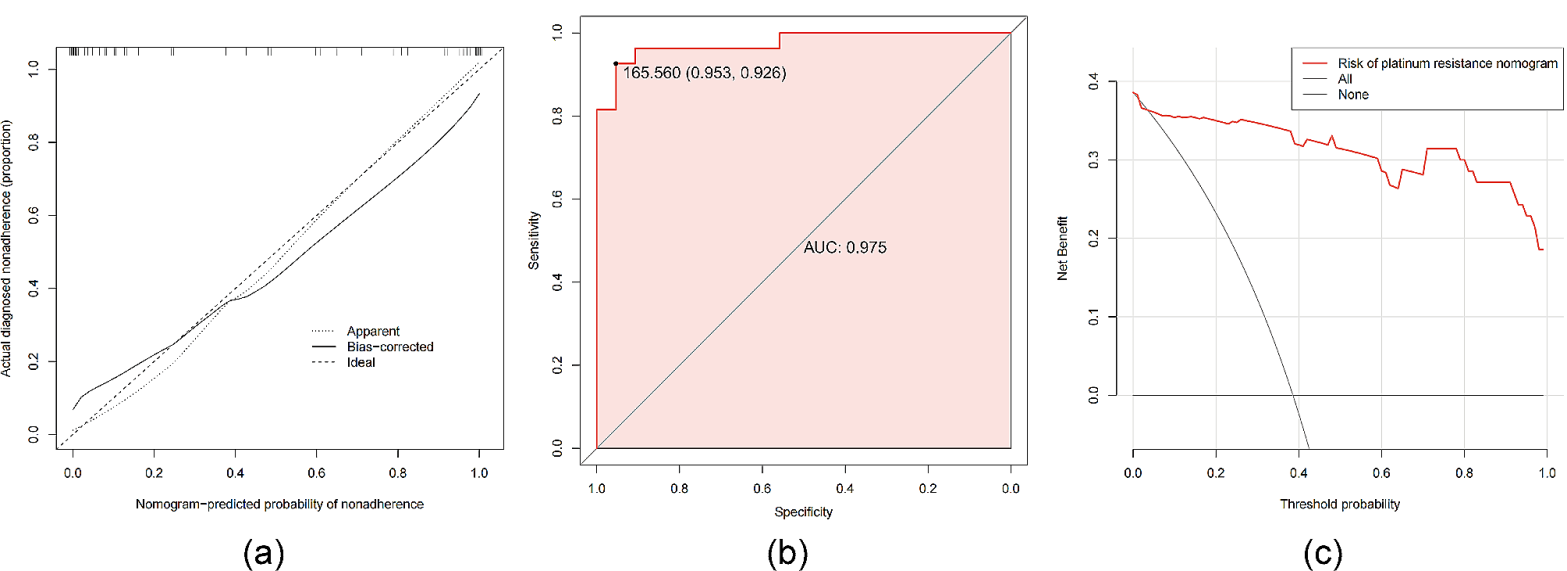
Predictive nomogram evaluation curve. (**a**) Calibration curve. (**b**) ROC curve. (**c**) Clinical decision curve.

The C-index of the nomogram constructed in this study was 0.975(95% CI: 0.9402-1). The model calibration curve showed that the apparent curve fits well with the curve after bias correction, indicating that the prediction model had a high degree of agreement (Fig. 6a). The cut-off value of total points in the computed nomogram of the ROC curve (Fig. 6b) is 165.6, the sensitivity of the prediction model was 92.59%, the specificity was 95.35%, and the decision curve maximum clinical net showed predictive benefit was within the threshold of 1-100%(Fig. 6c).

### External validation of platinum resistance prediction nomogram in epithelial ovarian cancer

**Fig. 7:**
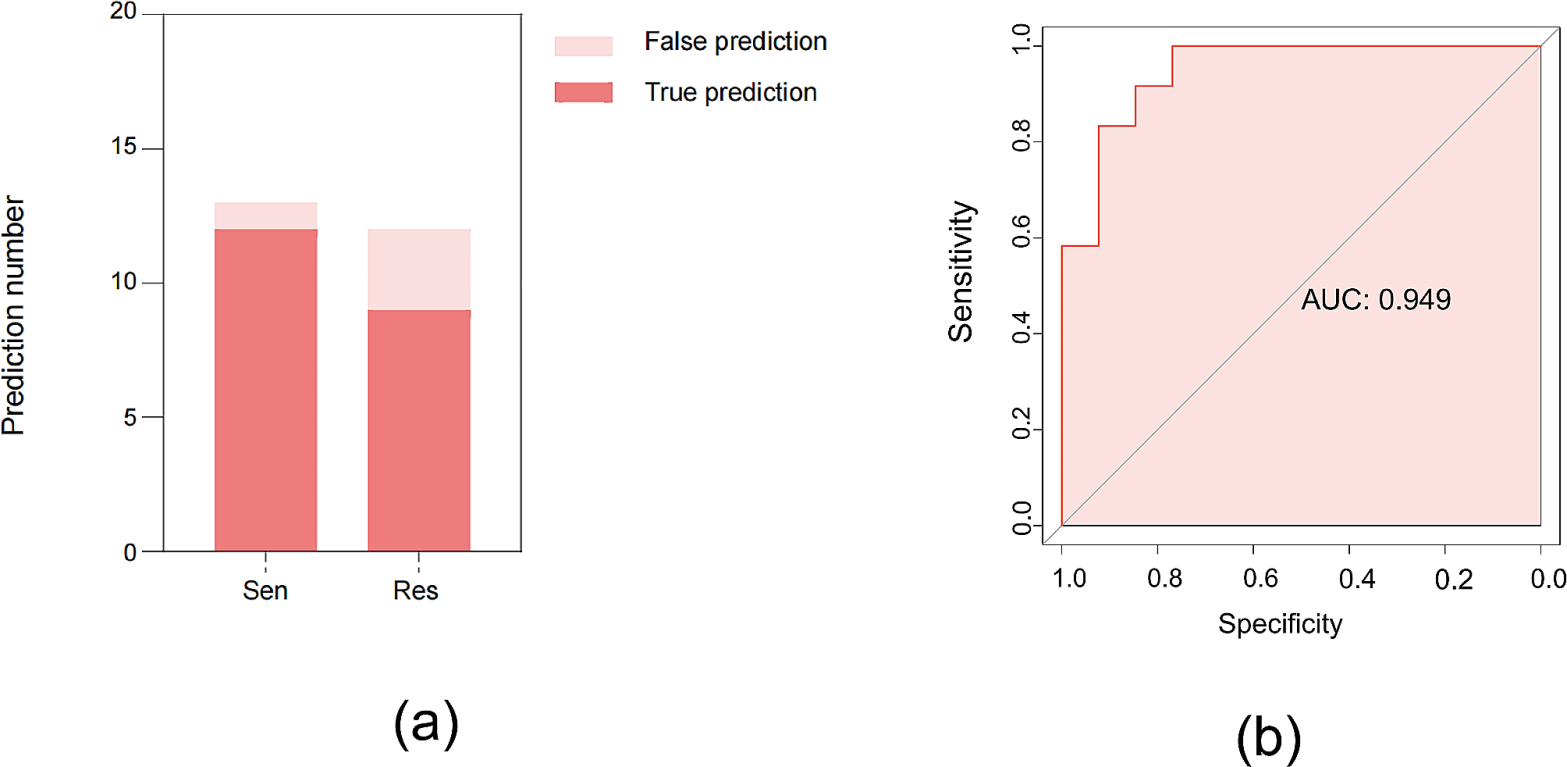
Nomogram of platinum resistance prediction in epithelial ovarian cancer validated by test cohort. (**a**) Comparison of prediction and actual situation. (**b**) ROC curve of test cohort.

All predictive variables of 25 patients in the test cohort were shown in Table 3. According to the optimized prediction model of platinum resistance in epithelial ovarian cancer, 25 patients in the test set were scored and predicted, and the results showed that the coincidence rate of nomogram prediction of platinum resistance was 84%(Fig. 7a). The Kappa of external validation of the prediction nomogram was 0.677, indicating strong consistency. The ROC curve (Figur 7b) showed that the AUC of the prediction model was 0.949, which proved that the nomogram still had good discrimination ability in the test set.

**Table 3:** Characteristics of patients in the training cohort (*n* = 70)

Variable		Sen-group(*n* = 13) (n(%))/$\bar {x} \pm s$	Res-group(*n* = 12) (n(%))/$\bar {x} \pm s$	p
HE4(pmol/L)		451.23 ± 414.143	849.73 ± 563.42	0.054
Maximum tumor diameter	≤ 5 cm	2(15.4)	3(25.0)	0.0548
	> 5 cm	11(84.6)	9(75.0)	
Vascular cancer thrombus	no	11(84.6)	8(66.7)	0.294
	yes	2(15.4)	4(33.3)	
Ascites cancer cell	no	9(69.2)	2(16.7)	0.011
	yes	4(30.8)	10(83.3)	
Neoadjuvant chemotherapy	no	11(84.6)	5(41.7)	0.025
	yes	2(15.4)	7(58.3)	
IL-6 IHC score	0	11(84.6)	4(33.3)	0.008
	1+	2(15.4)	6(50.0)	
	2+	0(0.0)	2(16.7)	
CXCL1 IHC score	0	11(84.6)	6(50.0)	0.062
	1+	2(15.4)	5(41.7)	
	2+	0(0.0)	1(8.3)	
CXCL2 IHC score	0	11(84.6)	5(41.7)	0.023
	1+	2(15.4)	5(41.7)	
	2+	0(0.0)	2(16.7)	
ABCC1 IHC score	0	11(84.6)	6(50.0)	0.069
	1+	2(15.4)	6(50.0)	
LRP IHC score	0	10(76.9)	3(25.0)	0.008
	1+	3(23.1)	7(58.3)	
	2+	0(0)	2(16.7)	
BCL2 IHC score	0	12(92.3)	4(33.3)	0.003
	1+	1(7.7)	6(50.0)	
	2+	0(0.0)	2(16.7)	

*p* < 0.05 The difference was statistically significant

## Discussion

Platinum-based combination chemotherapy is a necessary adjuvant therapy for epithelial ovarian cancer. However, about 20–30% of epithelial patients will primarily resist chemotherapy after initial chemotherapy, and the remaining patients will relapse in the long term and eventually develop resistance [15]. Platinum resistance seriously affects the prognosis of patients. Finding the predictive markers or characteristic factors of drug resistance in ovarian cancer is essential [16]. It is of great clinical significance for the early detection of platinum-resistant patients, alerting patients to chemotherapy response, and timely changing chemotherapy drugs to develop personalized treatment.

This study used the clinical data of epithelial ovarian cancer patients and the results of platinum resistance genes detected by immunohistochemistry of tumor tissues to construct a platinum resistance prediction model nomogram. Finally, the immunohistochemical scores of CXLC1, CXCL2, IL6, ABCC1, LRP, BCL2, vascular tumor thrombus, ascites cancer cells, maximum tumor diameter, neoadjuvant chemotherapy, and HE4 were employed. When evaluating the predictive value of the nomogram, the AUC reached 0.975, and the sensitivity and specificity were 92.59% and 95.35%, respectively Additionally, the external validation shows a significant coincidence rate, indicating a higher clinical application potential for the epithelial ovarian cancer prognostic nomogram.

In the nomogram, vascular tumor thrombus, ascites cancer cells, maximum tumor diameter, neoadjuvant chemotherapy, and HE4 were screened as clinical characteristics. Previous studies have also supported the predictive value of these clinical features. In other studies, vascular tumor thrombus has also been considered an influential factor affecting platinum resistance in epithelial ovarian cancer. Intravascular tumor thrombus, including lymphatic and vascular tumor thrombus, is closely related to tumor invasion and metastasis [17]. Cancer cells in ascites suggest a more significant tumor burden and malignant ascites contribute to ovarian cancer metastasis [18]. The particular microenvironment provided by ascites cancer cells and the influence of non-tumor cells in ascites may change the responsiveness of chemotherapy [19, 20]. For patients with advanced extensive metastasis, neoadjuvant chemotherapy can effectively reduce the volume of tumor lesions and tumor adhesions, thereby improving the surgical resection rate and reducing surgical complications [21]. However, in recent years, some randomized controlled trials and meta-analyses have suggested that neoadjuvant chemotherapy does not improve the survival benefit of patients. Some retrospective studies have observed a higher rate of platinum resistance in patients after neoadjuvant chemotherapy [22]. Neoadjuvant chemotherapy could change tumor gene expression, such as membrane transport-related protein down-regulation, and would enhance ovarian cancer cells’ dryness [1, 22, 23]. The tumor marker HE4 is vital for diagnosing and monitoring epithelial ovarian cancer. Primary research results have shown that HE4 is associated with the chemotherapy response of ovarian cancer [24, 25]. Clinical studies have also shown that serum HE4 levels are associated with platinum resistance in epithelial ovarian cancer [3].

The nomogram embracing platinum resistance genes IHC score of CXCL1, CXCL2, IL6, ABCC1, LRP, BCL2. Among them, the research results of CXCL1 and CXCL2 in epithelial ovarian cancer and other tumors have shown that they are related to the malignant biology of the tumor, and they have higher regression coefficients in the model, which has the most significant impact on the prediction model [26–28]. They have also fully demonstrated that chemokines are essential in platinum resistance in ovarian cancer. Related studies on ovarian cancer also show that IL-6 mediates tumor cell proliferation and invasion [29]. IL-6 can activate signaling pathways that lead to tumor proliferation, such as JAK-STAT3, associated with tumor cell proliferation and resistance to chemotherapy. ABCC1 is a basolateral transporter, which could excrete drugs into bile intestine and finally eliminate them from the body. Another study has shown that ABCC1 is a predictive marker of chemotherapy response in epithelial ovarian cancer [30]. In tumor chemotherapy, LRP could prevent drugs from entering cells through nuclear pores and exocytosis [31]. Studies related to drug resistance in ovarian cancer have also shown that LRP could predict drug resistance in ovarian cancer [32]. Bcl-2 family plays an essential role in apoptosis, an important molecule to regulate apoptosis and mitochondrial apoptosis. It regulates the intrinsic apoptosis pathway by neutralizing mitochondrial permeability molecules and is closely related to chemotherapy resistance of epithelial ovarian cancer [33]. Some drugs targeting Bcl-2 family members have shown efficacy in overcoming chemoresistance in ovarian cancer [34]. All the above studies showed that the genes the nomogram included were associated with platinum resistance in epithelial ovarian cancer at the cellular and molecular biological levels.

Previous studies have analyzed the predictive value of tumor gene characteristics for platinum resistance in epithelial ovarian cancer. Nevertheless, no research had assessed a model that predicts platinum resistance by integrating clinical traits and tumor gene properties. In this study, we identified clinical features and gene markers associated with platinum resistance in epithelial ovarian cancer and integrated the predictive factors of the two types of features to construct a prediction model for platinum resistance. The nomogram was utilized to better support clinical application, and the model’s evaluation revealed that it had a good predictive value. In the meantime, it is not too difficult to gather information on the clinical features and pathological examination results of patients, and using these data to assess patients’ drug resistance has good clinical operability. At the same time, we acknowledged that before being used in a clinical setting, the platinum resistance prediction model created in this investigation still needed to be further refined and validated in a multi-center study with a bigger sample size because of the limited sample size.

## Conclusion

We developed an operable nomogram for predicting platinum resistance in epithelial ovarian cancer based on clinical features and platinum resistance gene IHC score was evaluated by various methods. This nomogram could help clinical oncologists identify patients who are not responding to first-line platinum-based chemotherapy medications and help them create personalized treatment plans. After that, to further improve the model’s suitability for clinical usage, a multi-center cohort and a large sample size are necessary.

## Data Availability

The data is available from the corresponding author on reasonable request.
